# Engineered Polystyrene-Based Microplastics of High Environmental Relevance

**DOI:** 10.1021/acs.est.1c02196

**Published:** 2021-07-22

**Authors:** Amit Kumar Sarkar, Andrey Ethan Rubin, Ines Zucker

**Affiliations:** † School of Mechanical Engineering, Faculty of Engineering, Tel Aviv University, Tel Aviv 69978, Israel; ‡ Porter School of the Environment and Earth Sciences, Faculty of Exact Sciences, Tel Aviv University, Tel Aviv 69978, Israel

**Keywords:** degradation mechanism, fragmented morphology, environmental plastic, plastic model, particle size

## Abstract

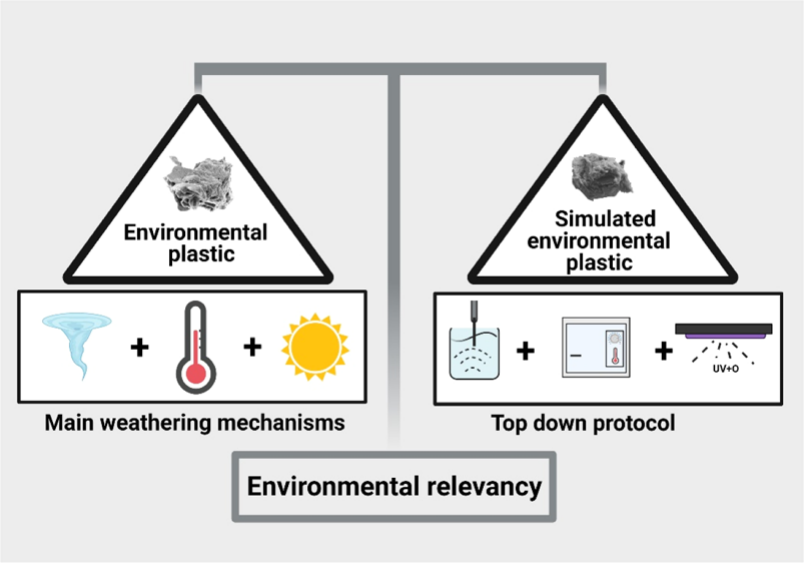

Microplastic (MP) pollution—an emerging environmental challenge of the 21st century—refers to accumulation of environmentally weathered polymer-based particles with potential environmental and health risks. Because of technical and practical challenges when using environmental MPs for risk assessment, most available data are generated using plastic models of limited environmental relevancy (i.e., with physicochemical characteristics inherently different from those of environmental MPs). In this study, we assess the effect of dominant weathering conditions—including thermal, photo-, and mechanical degradation—on surface and bulk characteristics of polystyrene (PS)-based single-use products. Further, we augment the environmental relevance of model-enabled risk assessment through the design of engineered MPs. A set of optimized laboratory-based weathering conditions demonstrated a synergetic effect on the PS-based plastic, which was fragmented into millions of 1–3 μm MP particles in under 16 h. The physicochemical properties of these engineered MPs were compared to those of their environmental counterpart and PS microbeads often used as MP models. The engineered MPs exhibit high environmental relevance with rough and oxidized surfaces and a heterogeneous fragmented morphology. Our results suggest that this top-down synthesis protocol combining major weathering mechanisms can fabricate improved, realistic, and reproducible PS-based plastic models with high levels of control over the particles’ properties. Through increased environmental relevancy, our plastic model bolsters the field of risk assessment, enabling more reliable estimations of risk associated with an emerging pollutant of global concern.

## Abstract 2

Microplastics (MPs) are emerging contaminants, posing a threat to the environment and human health. This research focuses on the synthesis of engineered MPs of high environmental relevancy and suggests them as an improved and controllable model for plastic risk assessment. Engineered MPs were designed through a synergetic top-down synthesis protocol. Accelerated thermal, photo-, and mechanical degradation procedures were applied. The engineered MPs had rough, oxidized surfaces with a fragmented morphology. Characteristics of engineered MPs were compared to those of environmental MPs. Engineered MPs were suggested as an improved model for plastics’ risk assessment.

## Introduction

Since its discovery in the 19th century, plastic has become an integral part of our modern lives. Plastics are typically composed of polymers—such as polyethylene (PE), polypropylene (PP), polystyrene (PS), and polyethylene terephthalate (PET)^1,2^ —and additives (e.g., colors and fillers) intended to enhance polymer properties and prolong their life.^3^ Associated with the plastic revolution is an increasing growth rate of plastic waste worldwide,^4^ which eventually reaches the oceans via inland waterways, wind, and tides.^5^

By 2050, accumulation of plastic waste in landfills and aquatic environments is estimated to reach 33 billion tons.^4^ This pervasive and persistent magnitude of plastic waste—and especially single-use plastic products—in the aquatic environment suggests plastic as a large-scale emerging environmental pollutant.^6^

**Figure 1:**
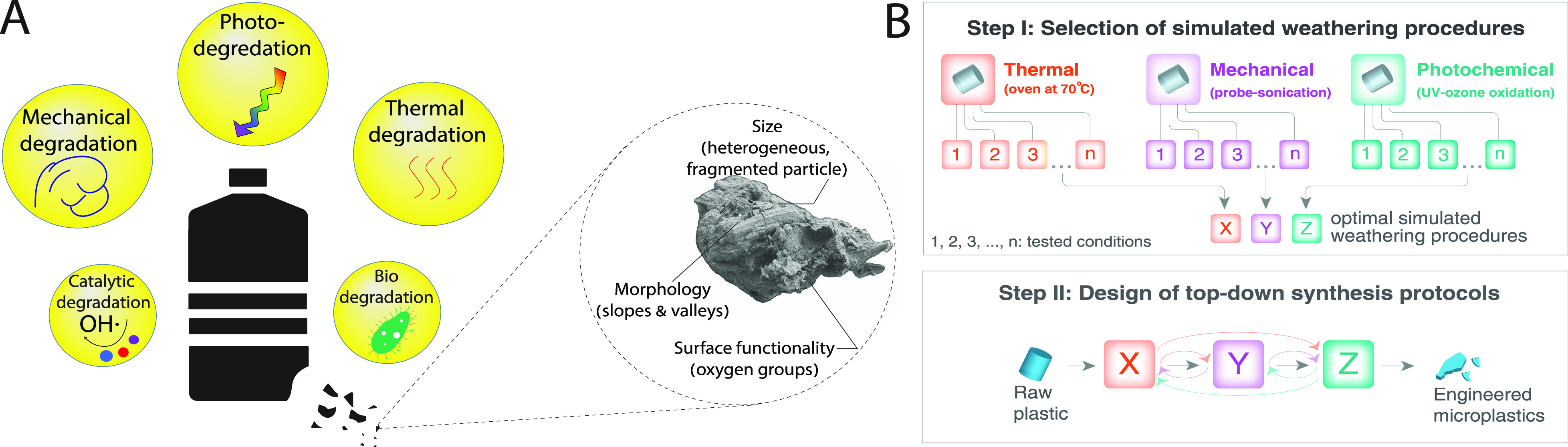
(A) Environmental degradation processes of plastic (left) and corresponding plastic product properties (right). (B) Schematic illustration of engineered MP design, including selection of individual simulated weathering procedures (top) and top-down synthesis protocols (bottom).

Rather than degrading quickly, plastics are susceptible to slow weathering processes,^7^ which in the aquatic environment include UV-driven photo-degradation (i.e., oxidation),^8^ wave-driven fragmentation,^9^ and thermal degradation^9^ (Figure 1A).^10^ Catalytic degradation (i.e., radical propagation) was also reported to occur under all weathering processes.^9^ These combined weathering processes result in plastic decomposition to micrometer (μm) and nanometer (nm) particles [microplastics (MPs) and nanoplastics (NPs), respectively]^11^ along with release of additives from the bulk plastics during decomposition.^3,12^

The interactions of plastic and biological substances in the aquatic environment influence its weathering.^13^ On one hand, microbial colonization on plastic surfaces (as biofilms) may form protective layers on the plastic surface and thus reduce photo-degradation.^14^ On the other hand, colonization by microorganisms may increase the probability of plastics being biodegraded, depending on the composition and structure of the microbial communities as well as the bioavailability of the synthetic plastic.^15,16^ Overall, although biofilm-plastic interactions are increasingly reported to have a role in the formation of MPs, such interactions are rather complex, diverse, and site-specific and not fully characterized.^17^

Weathered MPs—often characterized by rough, oxidized surfaces and heterogeneous fibrous or fragmental morphology—pose major environmental concerns due to their unknown fate and transport as well as their potential toxicity toward living organisms. Therefore, a massive research effort has been applied in recent years to MPs’ environmental implications throughout their life cycle, which have been found to be highly dependent on their physicochemical characteristics influenced by environmental weathering.^18−20^ For example, sorption levels of micropollutants onto MPs’ surfaces have been correlated with crystallinity and specific surface areas of MPs^21^ as well as functional groups and hydrophobicity of both MPs and micropollutants.^22−25^ The extent of overall deposition, retention, and transport of MPs in terrestrial systems^26^ was found to be dependent on MP density, shape, and size.^27^ MP toxicity, on the other hand, was found to change with MP size, specific surface area, and chemical composition.^28^

Use of real environmental plastic in studies exploring their environmental implications is difficult^29^ due to environmental plastics’ complex extraction and characterization techniques, potential contamination with non-environmental plastics during handling,^30^ and heterogeneous composition and size distribution (which hampers reproducible, statistically significant results). Because of these restraints as well as the fact that environmental implications of MPs are becoming a prominent field of research, most of the available data in the field are rapidly generated through controlled experiments using plastic models.^31,32^ Because physicochemical characteristics drive the fate, transport, and risk, such plastic models should accurately reflect the characteristics of environmental plastics while eliminating factors interfering with controlled risk assessment such as the wide size and compositional distributions of plastic particles.

Many MP models attempt to mimic environmental MPs, albeit insufficiently. A widely used model representing environmental MPs is commercially available polymeric microbeads,^33−39^ but these commonly used spherical microbeads are not accurate representations of secondary environmental MPs due to the microbeads’ smooth surfaces, homogeneous spherical shape, and pure polymeric composition.^40^ Another model used in literature is shredded (environmental) plastic debris, but this model results in limited reproducibility of results due to this technique’s limited control over polymer types and the presence of contamination from the field.^41^ Prospective top-down approaches have been suggested in literature, such as the use of commercial weathering chambers^42^ or application of single degradation mechanisms^43−45^ to degrade plastic products. However, the focuses of these studies were MPs’ risk toward the environment rather than verification of the resultant plastic particles’ resemblance to their supposed environmental counterparts.^45^ Overall, because inappropriate or unverified MP models may enable incorrect conclusions as to MPs’ interaction with their environment, there is an urgent need to fundamentally develop simulated MPs with environmentally relevant characteristics better representing those of their real environmental samples.

In this study, we use PS-based single-use products as raw plastic for top-down fabrication of environmentally relevant plastic particles (Figure 1B). We screen through simple lab procedures to simulate dominant weathering mechanisms and degrade the PS-based plastic in a systematic and environmentally relevant manner. Upon selection and optimization of individual weathering procedures—based on similarity of the resultant plastic particles to environmentally degraded plastics—we combine the most representative procedures into several, accelerated multi-stage protocols producing engineered plastic particles. We compare major physicochemical characteristics of our simulated engineered MPs to those of commonly used primitive models (i.e., microbeads) and semi-environmental plastics (i.e., same PS-based product placed under real environmental conditions). Our results suggest that the engineered MPs have comparable surface morphology and functionality to those of naturally weathered plastic particles, with a defined average particle size of 1–3 μm. Overall, this work offers a facile and reproducible method to fabricate plastic particles of higher environmental relevance than currently available plastic models while allowing control over particle characteristics. In the future, this tunable MP synthesis method could be applied to other plastic types, pushing the entire field of plastic pollution risk assessment toward higher reliability and real-world relevance.

## Materials and Methods

### Pretreatment of Raw-Plastic Particles

PS-based single-use forks were crushed and milled using a mechanical grinder (Ika Werke M20, Germany). The ground plastic particles were sieved using a stainless steel 100 μm mesh strainer. The sieved product (<100 μm) was used as raw plastic for all degradation procedures conducted in this study. The average size of the raw-plastic particles was ∼90–100 μm (Figure S1 in the Supporting Information). Raw-plastic particles were stored in a glass refrigerated container until use.

### Accelerated Oxidation Procedure

Oxidation of raw-plastic particles was conducted through exposure to UV and ozone in wet conditions (both in the absence and presence of salts) as well as in dry conditions. In wet conditions, 100 mg of raw-plastic particles was placed in a glass Petri dish and dispersed in 8 mL of deionized (DI) water from a Milli-Q ultrapure water purification system (Millipore, Billerica, MA) or salty water DI water with 33 g/L Red Sea premium salts, Red Sea Fish, Israel). Ethanol (EtOH) was added (20%) to allow increased dispersion of the hydrophobic raw plastic. Dry (100 mg) and wet samples were placed in a UV-Ozone (UV-O) chamber (ProCleaner, BioForce Nanosciences, USA) for a predetermined period (1–4 h). The spectral distribution of the UV irradiation is shown in Figure S2. The oxidized plastic particles were mixed following each exposure hour. The wet treated particles were filtered prior to analysis [other than wet analyses such as particle size distribution (PSD) and zeta potential] using a 0.45 μm nylon filter and washed with excess DI water. To assure that the filter paper does not influence the collected plastic particles (through contribution of additional nylon particles, e.g.), a control experiment was conducted in which only DI water was filtered (rather than degraded PS particle suspension). The negligible presence of particles in the retentate and filtrate confirmed that the filtration does not interfere with analysis of the degraded PS particles (Figure S3). The treated oxidized samples were collected using the nylon filter paper in solid form, transferred into glass vials, and stored in a refrigerator for further analyses.

### Accelerated Mechanical Procedure

Mechanical degradation of the raw-plastic particles was conducted using probe sonication (QSonica, LLC Q 125, USA) across different sonication times in an ice bath. First, 500 mg of raw-plastic particles was dispersed in 50 mL of DI water with 20% EtOH and poured into a 100 mL glass bottle. Sonication was conducted under sequences of 7 s on (70% amplitude) and 3 s off for 10, 20, or 30 min. The sonicated suspension was collected, filtered (using a 0.45 μm nylon filter), and refrigerated in glass vials for further analyses.

### Accelerated Thermal Procedure

A lab oven (Carbolite, MRC, China) was used to simulate thermal effects on the physicochemical characteristics of PS-based plastic. Raw plastic (100 mg) was heated in the oven at 70 °C for either 6 or 12 h in both dry and wet (DI water with 20% EtOH) conditions. In wet conditions, the dispersion was sealed in a 50 mL Teflon-lined stainless steel autoclave prior to thermal treatment to eliminate evaporative effects. The treated plastic particles were collected, filtered (using a 0.45 μm nylon filter), and refrigerated in glass vials until further analyses.

### Design of Top-Down Synthesis Protocols Combining Accelerated Oxidative, Mechanical, and Thermal Degradation Procedures

The optimal accelerated procedures on raw plastic (based on physicochemical similarity to environmental samples) by means of oxidation, mechanical, and thermal degradation were used to design multi-stage sequenced protocols. Single-step procedures were combined into multi-step protocols applied on raw-PS-based plastic to achieve its cumulative degradation representing dominant weathering processes. Treated plastics were then characterized to elucidate the changes in physicochemical properties during the degradation procedures and to compare their physicochemical changes to those of environmentally weathered plastics.

### Semi-environmental Plastic and PS Microbeads

In order to compare our engineered MPs to their environmental counterparts, we generated PS-based semi-environmental plastic particles in controlled conditions. The milled and sieved PS-based raw plastic was dispersed in DI water, covered by a quartz window (to avoid contamination and to allow passage of UV irradiation to the suspension), and placed on a rooftop at Tel Aviv University (latitude 31.11264, longitude 34.80617) for 11 months from September 2019 to August 2020. The weathered wet sample was filtered (using a 0.45 μm nylon filter), collected in a glass vial, and refrigerated until use as a semi-environmental plastic reference to evaluate the environmental relevance of our engineered MPs. Non-functionalized PS microbeads were also purchased from Spherotech, Inc. USA for comparison to our engineered MPs and the semi-environmental sample.

### Characterization Techniques

Differential scanning calorimetry (DSC, Q-2000) was used to determine the glass-transition temperature (*T*_g_) of the raw-plastic particles. Heating and cooling were carried out in the temperature range of 25–100 °C with a fixed heating rate (10 °C/minute) under an inert nitrogen atmosphere. The raw plastic and treated plastic with individual simulated weathering procedures, surface chemistry, surface morphology and composition, and PSD were characterized by attenuated total reflection (ATR)-equipped Fourier transform infrared spectrometry (FT-IR), high-resolution scanning electron microscopy (HR-SEM) coupled with energy-dispersive X-ray spectroscopy (EDX), and micro-flow imaging technique, respectively.

FT-IR (Tensor 27, Bruker, USA) spectra were recorded in the range of 400–4000 cm^–1^ at a spectral resolution of 4 cm^–1^ with 16 scans. For each spectrum, approximately 10 mg of the dry sample was placed and compressed on the ATR and the spectrum was normalized to the background spectrum. The surface morphologies of raw plastic and treated plastic were examined using HR-SEM (ZEISS, Gemini SEM 300, Germany) in high vacuum mode and an acceleration voltage of 3 kV. All samples were sputter-coated (Quorum-150TS plus; UK) with Chromium for 5 min before HR-SEM imaging. EDX was used for elemental analysis of probe-sonicated plastics using a silicon drift detector-x-act from Quanta 200 FEG Environmental SEM. Particle sizes and concentrations were quantified by micro-flow imaging (PSD-MFI5200, CL, USA), wherein a digital camera with an illumination and magnification system captured in situ images of suspended particles in a flowing sample. Upon injection of the plastic particle suspension into the flow cell, the plastic particles are illuminated by a light-emitting diode (475 nm) and imaged using a digital camera. Each image was processed to determine the equivalent circular diameter of particles in the range of 1–100 μm.

Surface charges, surface crystallinities, and specific surface areas were also determined for the final engineered MPs, semi-environmental MPs, and PS microbeads. The zeta potentials (determining surface charge) of each sample were measured twice at 25 °C using a Zeta-Sizer system (Malvern Instruments, UK).^46^ The X-ray diffraction (XRD) patterns (determining crystallinity) of each sample were measured using an X’PERT system with Cu Kα radiation between 10° and 90° at a scan rate of 2° per min. Specific surface area (SSA) measurements were obtained using Brunauer–Emmet–Teller (BET) measurements on a Quantachrome Autosorb LX4, Austria, with krypton (Kr) as the analysis adsorptive. All samples were degassed under vacuum at 50 °C for 16 h before analysis to ensure moisture-free samples.

## Results and Discussion

### Effect of Individual Accelerated Degradation Procedures on the Properties of PS-Based Plastic

Top-down fabrication of environmentally relevant MPs in lab conditions should simulate the major weathering processes driving the degradation of bulk plastics in the environment. To ensure that the selected lab-based simulated procedures best represent natural weathering processes, changes in the physicochemical properties of the degraded plastics were compared to those observed in naturally weathered plastics.

### Photo-degradation

**Figure 2:**
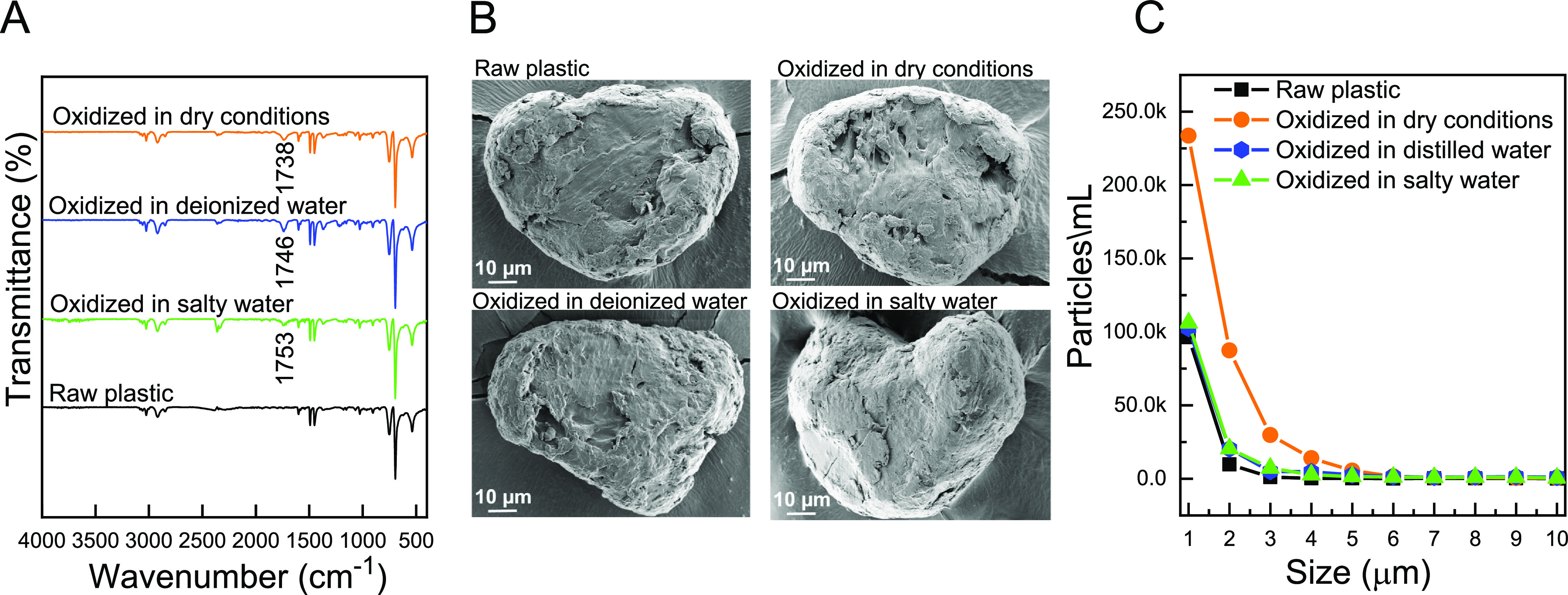
Characteristics of PS-based plastic particles following simulated photo-degradation procedures under a UV-O instrument for 3 h in dry and wet conditions (DI water and salty water at pH 8.5 and conductivity of 66.07 μS/cm). (A) FT-IR spectra, (B) representative HR-SEM images, and (C) PSD in the range of 1–10 μm. In wet experiments, 100 mg of raw-plastic particles was dispersed in 10 mL of aqueous solution containing 20% EtOH.

To mimic environmental photo-degradation, we exposed raw plastics to a UV-O instrument, which facilitated UV irradiation and oxidation via ozone and radical reactions. FT-IR spectra of the UV-O-treated plastics in all experimental conditions showed new peaks at approximately 1700 and 2300 cm^–1^, which were not observed in the spectra of raw PS-plastic (Figures 2A and S4). Whereas the peak at 2300 cm^–1^ is associated with CO_2_^47^ from measuring conditions, this new peak at ∼1700 cm^–1^ was due to the formation of carbonyl functional groups during UV-O treatment.^48^ Photo-degradation processes typically result in chain scission and cross-linking reactions, facilitating the formation of MPs^49^ with ketones.^48^ Such observations were reported for environmentally weathered PS-based plastics.^50−54^ It should be noted that the insertion of carbonyl functional groups was also reported following biodegradation.^14,15^ Thus, although biodegradation is excluded from our accelerated procedures, its associated surface functionalization is expressed in our degraded MPs. Irradiation time of 3 h was selected because of its highest relative peak intensity at ∼1700 cm^–1^ (which was similar to the relative peak intensity at 4 h of irradiation) in all tested conditions, including both wet (presence and absence of salt) and dry conditions (Figure S4).

HR-SEM images of large plastic particles were compared before and after photo-degradation (Figure 2B). Oxidized plastics in dry and wet conditions (in the presence and absence of salts) exhibited similar surface morphology to that of raw plastic, most likely because the long polymer chain responsible for structural integrity was unaltered in the tested conditions and the general morphology was therefore maintained. Nevertheless, we observed a change in the raw plastic color from white to yellow following the UV-O oxidation procedure (Figure S5), which has also been reported for the degradation of plastic in environmental conditions.^49^

The size distribution of suspended particles examined in the range of 1–10 μm was similar for all plastic samples (including raw plastic). Upon degradation, more than 99% of the particles were found to be smaller than 5 μm, and formation of 1–3 μm particles from the breakdown of larger particles could be detected. However, only a slight change in large particle concentration was observed following degradation. This trend is attributed to the fact that degradation of a few large particles translates to a large number of smaller particles. Particles smaller than 1 μm were below the measurement range of the analytical instrument and could not be quantified using the applied dynamic image analysis. Therefore, we focused the PSD discussion on taking the concentration of 1 μm of plastic particles and the particle size range of 1–10 μm.

The highest concentration of degraded 1 μm plastic particles was achieved in dry conditions (234k particles/mL of suspended sample), suggesting that dry plastics are more greatly impacted by the UV-O oxidation procedure compared to plastics in wet conditions (Figure 2C). This observation can be explained by the efficient light absorption in dry conditions compared to wet conditions where light scattering effects occur. Based on the changes in surface functionality and PSD in the range of 1–3 μm, we chose 3 h of dry irradiation as an optimal UV-O oxidation procedure.

### Mechanical Degradation

To mimic mechanical degradation of plastic in aquatic environments, raw-plastic suspensions were probe sonicated across different times. Under aquatic environmental conditions, shear forces (from waves and wind currents) enable the physical aging of plastic material through erosion,^55^ which promotes the formation of MPs.^56,57^ Specifically, mechanical forces can impose elevated friction on the surface of plastic against natural shoreline beach sediments.^46^

**Figure 3:**
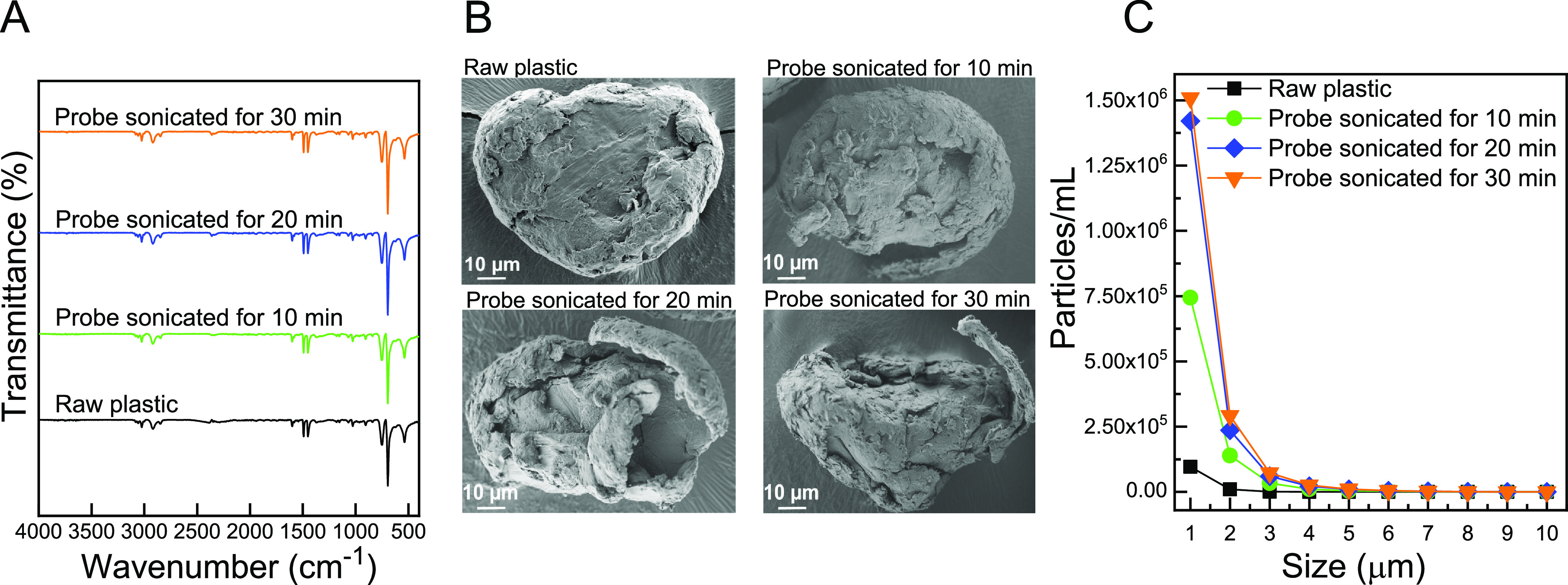
Characteristics of PS-based plastic particles following simulated mechanical degradation procedures in wet conditions (DI water and 20% EtOH) for 10, 20, and 30 min. (A) FT-IR spectra, (B) representative HR-SEM images, and (C) PSD in the range of 1–10 μm.

FT-IR analysis did not show any noticeable changes in surface functionality following sonication in all the tested conditions (Figure 3A). Unlike photo-degradation, sonication did impart significant changes in surface morphology as observed through HR-SEM. Following probe sonication, the surface morphology of the treated plastic was severely damaged^58^ in all three timelines examined, with noticeable changes in surface morphology appearing with long sonication times (Figure 3B). The obtained rough surface morphology of the treated plastics was similar to that of weathered PS-based plastics as reported in the literature.^59^Figure 3C shows that probe sonication for 20 and 30 min resulted in high concentrations of 1 μm particles (1.42 × 10^6^ and 1.50 × 10^6^ particles/mL, respectively), which was used as our direct indicator of levels of degradation, while 10 min of probe sonication resulted in a lower number of particles in the 1 μm range (7.44 × 10^5^ particles/mL).

The EDX of plastic particles formed after sonication showed the presence of carbon and oxygen elements (from plastic particles) as well as metallic elements, indicating sample contamination from abrasion of the probe tip during sonication (Figure S6). The abrasion from the probe tip at elevated sonication times was further confirmed by conducting EDX to control the sample of particles formed following sonication of DI water (i.e., in the absence of plastic particles), showing the presence of metallic particles composed of lead, titanium, iron, and aluminum originating from the probe alloy (Figure S7). As the probe sonication for 20 and 30 min had a high contamination rate (∼10% of particles) compared to 10 min of sonication (∼2.5%, Figure S8), we selected probe sonication for 10 min as the optimal accelerated mechanical procedure. Separation of the metal particles from the plastic suspensions can be further achieved by simple centrifugation.

### Thermal Degradation

**Figure 4:**
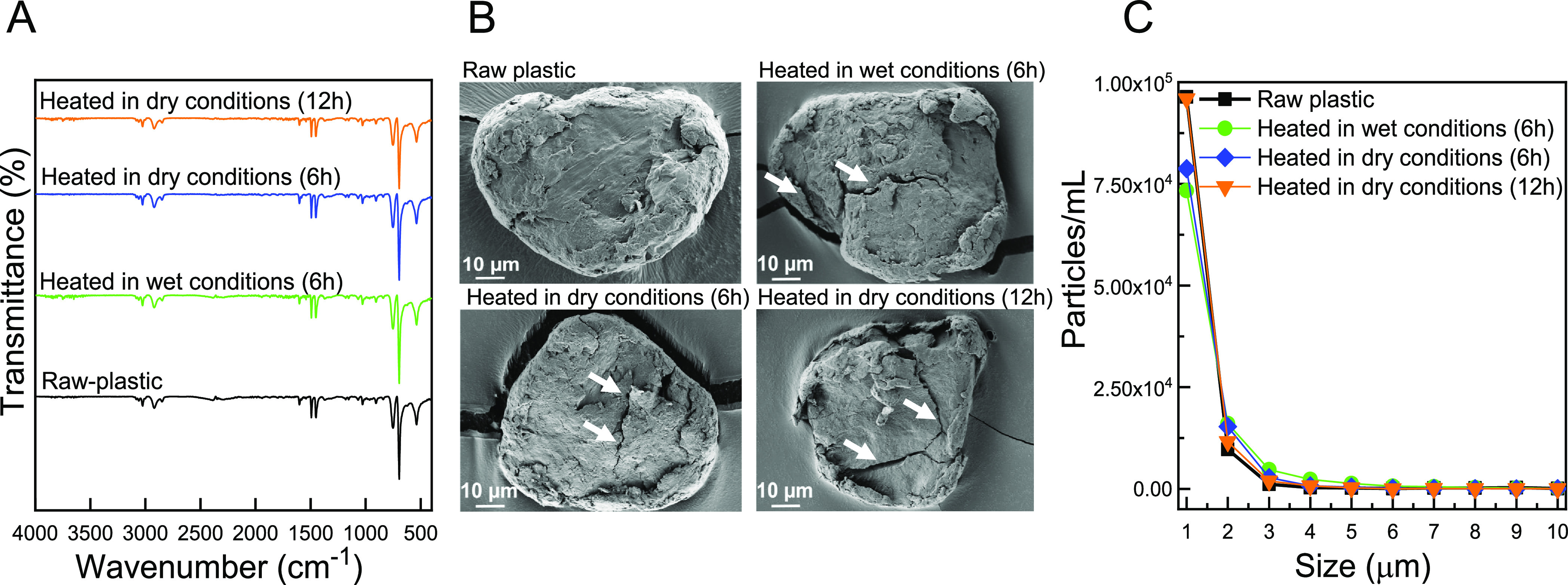
Characteristics of PS-based plastic particles following simulated thermal procedures in dry (6 and 12 h) and wet conditions (DI water and 20% EtOH; 6 h) (A) FT-IR spectra, (B) representative HR-SEM images, and (C) PSD in the range of 1–10 μm. Arrows in HR-SEM images represent cracks on the plastic surface.

The *T*_g_ of the raw plastic was determined to be 94.5 °C (Figure S9), suggesting that thermal treatment at temperatures lower than this *T*_g_ should prevent transition to a viscous or rubbery state of raw plastic. Therefore, we chose 70 °C for the accelerated thermal degradation of the raw plastic under dry and wet conditions. The FT-IR analyses did not show addition of new peaks, suggesting negligible surface functionalization during thermal treatment (Figure 4A). However, surface cracks (shown by arrows, Figure 4B) appeared after both six and 12 h of thermal treatment. Such observations in surface morphology were also reported for naturally weathered plastic surfaces.^60^ Crack formation most likely occurs during thermal treatment due to degassing of volatiles, which shrinks the plastic surface and promotes cracking from surface tensile stress.^61^

Figure 4C reveals a 1 μm particle concentration for plastics after 12 h of thermal treatment similar to that of raw-plastic particles, indicating the limited role of the applied thermal procedure in fragmentation of the plastic particles. However, the generated cracks on the plastic surface could predispose the particles to further fragmentation, especially under post-mechanical treatment. Thermal treatment for 6 h resulted in a slight decrease in the 1 μm particle concentration, possibly due to agglomeration of plastic particles, the effects from which were not offset by formation of new particles through fragmentation. Based on the noticeable morphological change, we chose 12 h (dry) of thermal treatment as an optimal thermal procedure.

### Effect of Combined Accelerated Degradation Procedures on the Properties of PS-Based Plastic

The optimal accelerated procedures for oxidation (3 h of dry UV-O oxidation procedure), mechanical degradation (10 min of probe sonication in 20% EtOH), and thermal (12 h at 70 °C) degradation were permuted to design a series of multi-stage protocols (Figure 1B). The three multi-stage protocols were (1) thermal > mechanical > oxidation; (2) thermal > mechanical > oxidation > mechanical; and (3) thermal > oxidation > mechanical. We selected thermal degradation as the first applied procedure because it results in rough and cracked surfaces, making the plastic more vulnerable to subsequent mechanical and oxidation procedures.

**Figure 5:**
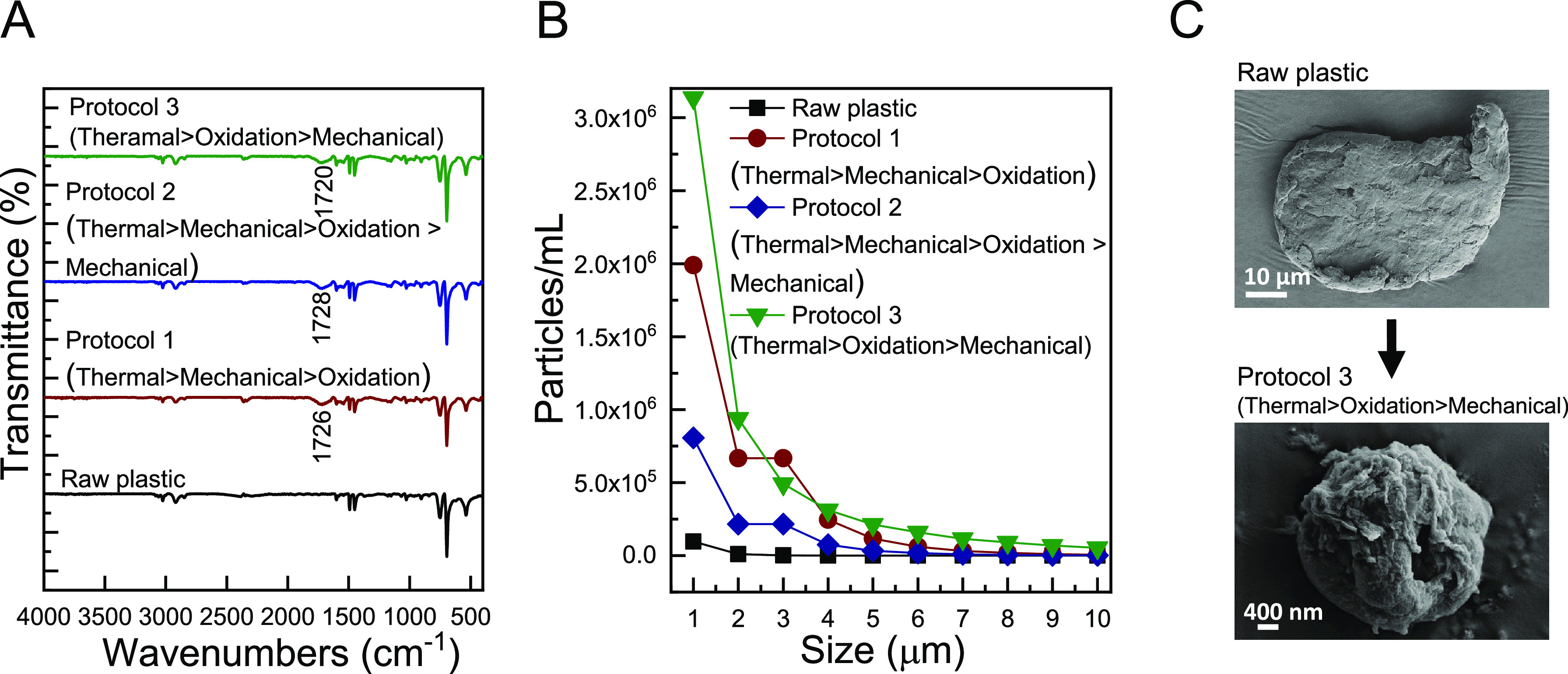
Characteristics of PS-based plastic particles subjected to top-down synthesis protocols that combine accelerated oxidation (3 h/dry, UV-O), thermal (12 h/dry, oven), mechanical (10 min/20% EtOH, probe sonicator) procedures (i.e., Protocol-3). (A) FT-IR (B) PSD in the range of 1–10 μm, and (C) and HR-SEM of representative raw plastic (left) vs preliminary engineered MPs (right).

The degraded plastics treated in all three protocols showed the characteristic FT-IR peak of carbonyl groups at ∼1700 cm^–1^ (Figure 5A), similar to the peak observed for the individual accelerated UV-O procedure (Figure 2A). Protocol-1 resulted in a higher concentration of 1 μm particles (1.9 × 10^6^ particles/mL) compared to each of the individual accelerated procedures (Figure 5B). Interestingly, once an additional probe sonication step was applied (i.e., Protocol-2), the concentration of 1 μm of particle counts dropped, probably due to breakage of particles into sizes below the measurement range. Protocol-3 resulted in the highest concentration of 1 μm of particles (3.2 × 10^6^ particles/mL) with a noticeable population of particles in the range of 2–4 μm (Figure 5B). Representative images of degraded plastic particles following Protocol-3 show severely damaged particle surfaces as compared to the smooth surfaces of the raw-plastic particles, together with a significant change in size (from 90 to 100 μm for raw-plastic particles to ∼1 μm for plastics treated by Protocol-3) (Figure 5C). To further examine the smaller particle size and damaged plastic surfaces in the overall population, we used ImageJ analysis on a series of HR-SEM images of raw plastic (Figure S1) and plastic treated by Protocol-3 (Figure S10). This analysis confirmed a clear shift of plastic particles’ range from ∼100 μm to below 1 μm. Based on degradation in particle size and the obtained change in surface functionality, Protocol-3 was selected as the most suitable protocol to produce engineered MPs. Moving forward, we extensively characterized the engineered MP product and compared its characteristics to those of both a PS microbead model often used for risk assessment and PS-based semi-environmental plastic.

### Comparison of Engineered MPs to the Polymeric Microbead Model and Environmental Plastic

The overall surface morphology of the obtained engineered MPs was fragmented, markedly different from the smooth, spherical morphology of the PS microbead particles but similar to environmental PS plastics (Table S1).^53,54,62,63^ The color of real environmental PS plastic appears to be yellow,^64^ similar to that of engineered MPs (Figure S5). An additional peak (i.e., not a pristine PS plastic peak) was reported in the range of 1650–1750 cm^–1^ for many extracted PS-based environmental MPs (corresponding to the insertion of oxygen functionalities due to environmental weathering), which also appeared for engineered MPs.^50−54,63^ We have also collected large PS-based MPs from the Israeli coast of the Mediterranean Sea (Tel Aviv, Israel), which had a similar fragmented and rough morphology with an oxidized surface (Figure S11).

However, bulk properties of environmental PS MPs are hard to assess due to the difficulty in collecting large volumes of environmental plastics of a single plastic origin. Additionally, surface impurities^50,51^ and heterogeneity in plastic sizes and aging periods^65^ also hinder the use of extracted MPs as a reference material for assessing the environmental relevance of engineered MPs.^66^

**Figure 6:**
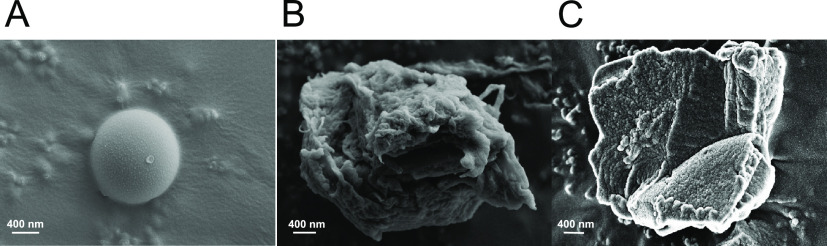
Representative HR-SEM images of (A) 1 μm of PS microbeads, (B) PS-based plastic particles subjected to top-down synthesis protocols that combine accelerated oxidation, thermal, mechanical procedures (Protocol-3), and (C) semi-environmental plastic (raw-plastic particles naturally weathered for 11 months on a Tel Aviv University rooftop).

Controlled aging of plastics may be the best available reference for comparison to engineered MP properties. Semi-environmental plastic may hold many advantages as a reference as (1) the raw plastic to be aged is identical to the one used to produce engineered MPs, (2) it is produced in a controlled environment, where aging time and aging conditions are known, (3) impurity content decreases and, (4) large volumes of MPs can be produced for bulk and surface analyses. For these reasons and as past studies used controlled simulated-PS^59,67,68^ as a standard reference to assess the bulk properties of MPs, we used semi-environmental plastic aged for 11 months in outdoor conditions (Figure 6) as our reference.

**Figure 7:**
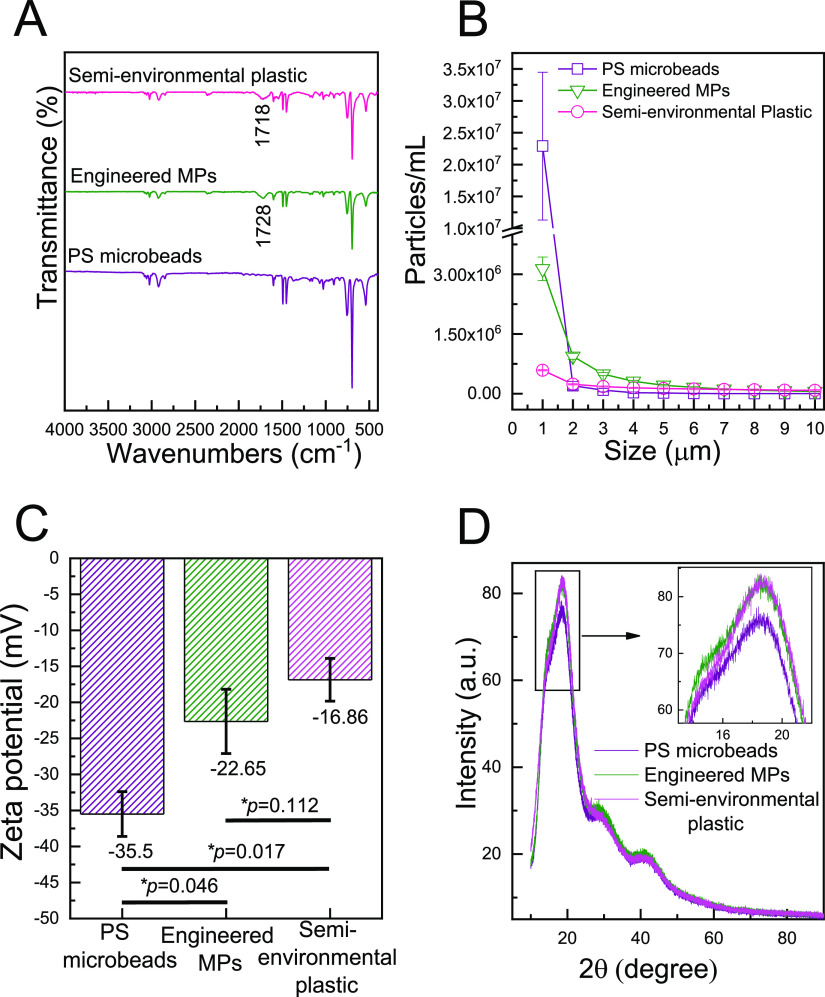
Comparison of characteristics of raw plastic, engineered MPs, and semi-environmental plastics by means of (A) FT-IR spectra, (B) PSD in the range of 1–10 μm, and (C) zeta potential, and (D) XRD (inset: zoom in to show similarity in peak intensity to engineered MPs and the semi-environmental sample).

The appearance of semi-environmental plastic is also yellow, similar to that of engineered MPs and real environmental PS plastic.^64^ The FT-IR patterns of both engineered MPs and semi-environmental plastic are similar in nature, with the emergence of the characteristic peak associated with carbonyl groups for both (Figure 7A). PS microbeads did not show any characteristic peak at 1700 cm^–1^, implying that the PS microbeads lack oxygen functionalities. To determine the aging time of the engineered MPs, we compared its normalized carbonyl index (CI)—defined as the integrated area ratio of the carbonyl bond to the carbon–carbon bond and often used as an aging index^69^ —to that of semi-environmental plastic. The calculated CI index suggests that the engineered MPs (Figure S12) produced through the accelerated protocol (roughly <16 h) was equal to 10–11 months of environmental weathering.

PSD analysis shows similar size distributions of the engineered MPs and the semi-environmental plastic; however, even with similar initial starting particle concentrations, the two degradation processes resulted in a significant difference in their resultant 1 μm particle concentrations (Figure 7B). This difference in final particle count could be attributed to the fact that the semi-environmental plastic was kept under sunlight irradiation with no external mechanical forces (which also hinder it from reaching true environmental realism), whereas the engineered MPs were subjected to both chemical and mechanical aging. The PS microbeads had a narrow PSD (single peak at 1 μm) compared to that of the semi-environmental plastic and the engineered MPs.

The surface charge of the synthesized engineered MPs was determined through zeta potential measurements and was compared to the surface charge of semi-environmental plastic and PS microbeads. The zeta potential of the engineered MPs product (−22.65 mV) was not statistically significantly different from that of the semi-environmental sample (−16.86 mV) but statistically significantly different than that of PS microbeads (*p* value: 0.046, Figure 7C). The similarity in zeta potential between engineered MPs and the semi-environmental sample could be attributed to similar physicochemical changes occurring in both the engineered MP product and semi-environmental plastic (e.g., weathering-induced carbonyl functional groups, which influence the surface charge).

Similar XRD spectra were observed for both the engineered MPs and the semi-environmental sample (Figure 7D), suggesting that they have similar crystallinities (determined by both the location and intensity of XRD peaks). However, lower crystallinity was observed for PS microbeads as compared to engineered MPs and semi-environmental plastic. Interestingly, the XRD spectra of raw plastics was also similar to that of engineered MPs (Figure S13), which can be explained by two contradictory processes occurring simultaneously. Photo-oxidation may increase crystallinity in polymers because the amorphous regions within the plastic are more susceptible to photo-degradation than those of crystalline regions.^70,71^ On the contrary, due to insertion of oxygen groups in the polymeric chains, amorphous or crystalline domains in polymer chains are interrupted, subsequently lowering the polymeric crystallinity.^72,73^ These contradictory effects may result in similar crystallinities when comparing degraded and non-degraded plastics.

Surprisingly, we noticed a smaller SSA for engineered MPs (0.379 m^2^/g) than those of raw plastic (0.581 m^2^/g) and semi-environmental plastic (0.618 m^2^/g), most likely because of the method by which oxygen surface functionalities were imparted on the engineered MPs during the accelerated photo-degradation procedure. Functionalization by carbonyl groups may occupy sites available for Kr adsorption during SSA measurements, thus resulting in a decreased SSA.^74^ The moderate oxygen functionalization occurring during environmental weathering in semi-environmental plastic, however, resulted in a negligible difference in the SSAs of raw plastic and semi-environmental plastic.

Altogether, the physicochemical characteristics of engineered MPs—including surface functionality, surface morphology, PSD, surface charge, and crystallinity—were comparable to those of the semi-environmental sample but inherently different from those of PS microbeads. The inherent differences between the characteristics of PS microbeads as compared to their environmental counterparts suggest that they are not an ideal model for environmental plastics, especially in fate, transport, and toxicity studies, which are driven by weathering and surface characteristics.^69^ For example, the presence of oxygen functionalities in weathered plastic may alter its interactions with surrounding substances^59^ and generation of asymmetric rough morphologies of weathered plastics can affect the toxicity^67^ and transport phenomenon.^59,71^

In this study, weathering processes are mimicked through lab-simulated techniques in such a way that they combine maximal effects of high environmental relevance. Of particular note, the engineered MPs were synthesized over 10 times with consistent similarity to the semi-environmental sample (demonstrated in PSD error bars and relative standard deviation of 7.4–9.4%, Figure 7B), indicative of the high reproducibility and reliability of the engineered MPs. Therefore, our engineered MPs bolster its relevance as a more robust representative of environmental MP in controlled risk assessment.

We calculated the energetic efficiencies per particle produced of single degradation procedures and compared them to the overall energetic efficiency of Protocol-3 (detailed calculation in the Supporting Information) to learn about the synergistic effects which occur through the combination of several degradation mechanisms. Using equations S1–S3, we showed that the probe sonication procedure was the most energy-efficient procedure requiring 1.66 J/L per particle produced. UV-O treatment in the selected conditions (i.e., 3 h of dry treatment) requires 76.05 J/L per particle produced, while thermal treatment requires 540 J/L per particle produced. The combined top-down synthesis protocol had a lower energy consumption (0.37 J/L per particle produced) compared to individual procedures, which was also 5 times lower than that of the most efficient procedure (i.e., probe sonication). This energy calculation suggests that combination of all three procedures into one protocol is critical not only to increase environmental relevance but also to enable a synergistic effect and decrease the energy consumption of the overall process.

The suggested method provides high levels of control over the model particles’ properties and thus could be tuned to produce PS-based engineered MPs of various sizes (e.g., NPs vs MPs) or age by altering the accelerated weathering procedures (e.g., degrading at various temperatures and sonication intensities). In fact, these engineered MPs can be (post) modified through sorption of typical co-existing substances—such as biomacromolecules (i.e., eco-corona) and micropollutants—to evaluate their role in the behavior of MPs in the aquatic environment. Furthermore, the protocol can form the basis for the future development of engineered MPs derived across polymer types (e.g., PET or PP-based MPs, which are abundant in the aquatic environment). We outlined the design, fabrication, and characterization of the first prototype of an environmentally relevant PS-based plastic model, which can be ultimately used as an improved model for future risk assessment studies as well as general standardized MPs of high environmental relevance. In future studies, the shelf-life of engineered MPs as well as their performance in fate, transport, and toxicity assays as compared to environmental plastics should be determined to validate the hypothesis claiming engineered MPs in this work as an emerging, highly representative plastic model augmenting the field of risk assessment.

## Supporting Information Available

PSD for raw plastic; spectral distribution of UV irradiation; negligible particle contamination during filtration; functionalization following UV-O treatment across various conditions; change in color following UV-O treatment; elemental analysis of particles obtained following probe sonication; particle count of probe sonicated solutions in the presence and absence of plastics; DSC spectrum of raw plastic; PSD for engineered MPs; FT-IR spectra and their corresponding sizes, and digital images of real PS-based environmental plastic collected from the Israeli coast of the Mediterranean Sea, Tel-Aviv, Israel; FT-IR spectra and calculation of CI; XRD spectra of raw plastic and engineered MPs; and characteristics of extracted PS-based environmental MPs (PDF)
