# Supplementary material for: Engineered Polystyrene-Based Microplastics of High Environmental Relevance
Source: Environ Sci Technol. 2021 Jul 22;55(15):10491–501. doi: 10.1021/acs.est.1c02196 (PMC8383278; doi:10.1021/acs.est.1c02196)
Supplement: Supplementary file 1 — es1c02196_si_001.pdf [file es1c02196_si_001.pdf]

Supporting Information

Engineered Polystyrene-Based Microplastics of High Environmental Relevance

Environmental Science and Technology

Amit Kumar Sarkar [1,2†], Andrey Ethan Rubin [2†], Ines Zucker [1,2*]

[1]School of Mechanical Engineering, Faculty of Engineering, Tel Aviv University, Tel Aviv 69978, Israel

[2]Porter School of the Environment and Earth Sciences, Faculty of Exact Sciences, Tel Aviv University, Tel Aviv 69978, Israel

[†] Co-first authors

* Corresponding author; Address: Tel Aviv University, Tel Aviv 69978, Israel; Tel: (+972) 73-3804581; email: ineszucker@tauex.tau.ac.il

Contains 13 Figures, one Table, and a calculation of energy requirements.

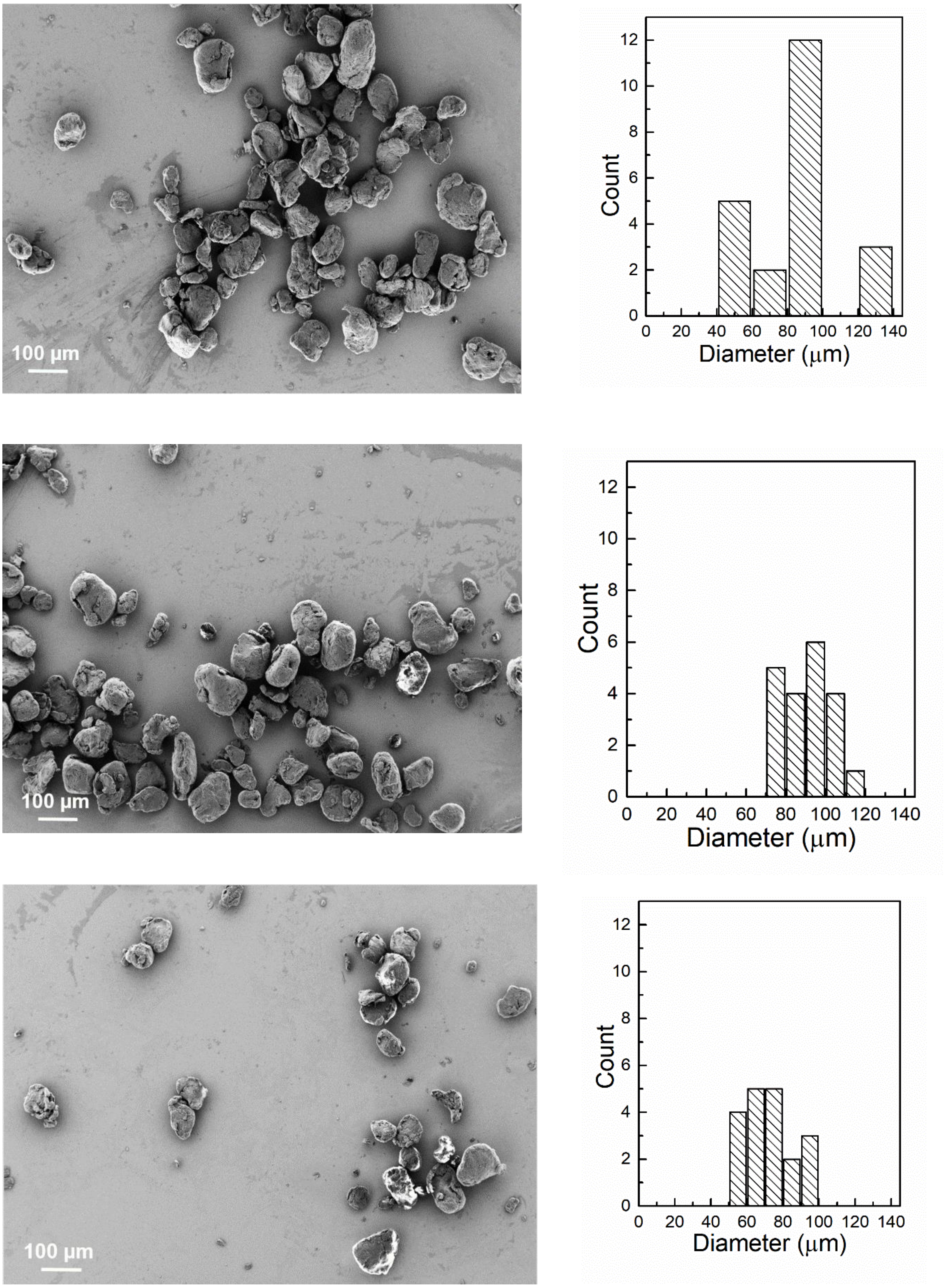

Figure S1. HR-SEM images of raw plastic and their corresponding particle size distributions calculated using the ImageJ postprocessing software.

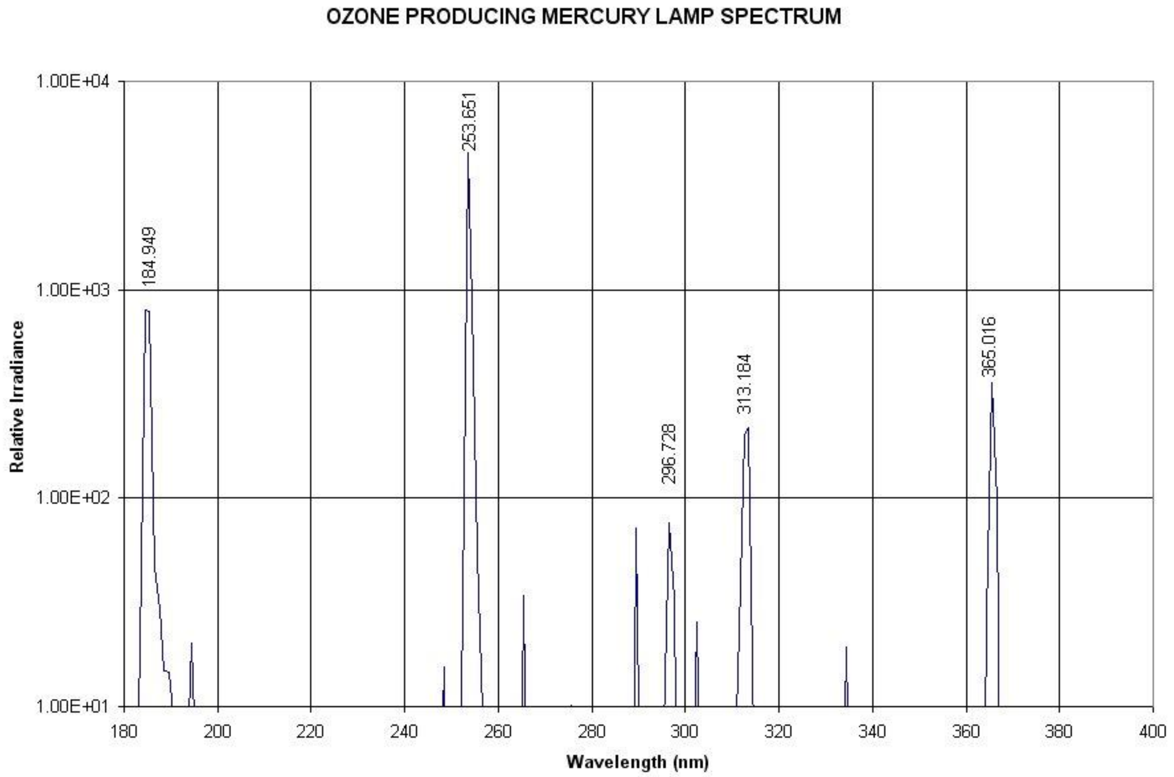

Figure S2. The spectral distribution (received from ProCleaner, Bioforce Nanosciences, USA) of UV irradiation in the UV-O chamber used for photo-degradation experimentation.

Scale bar: 100 µm	Scale bar: 10 µm

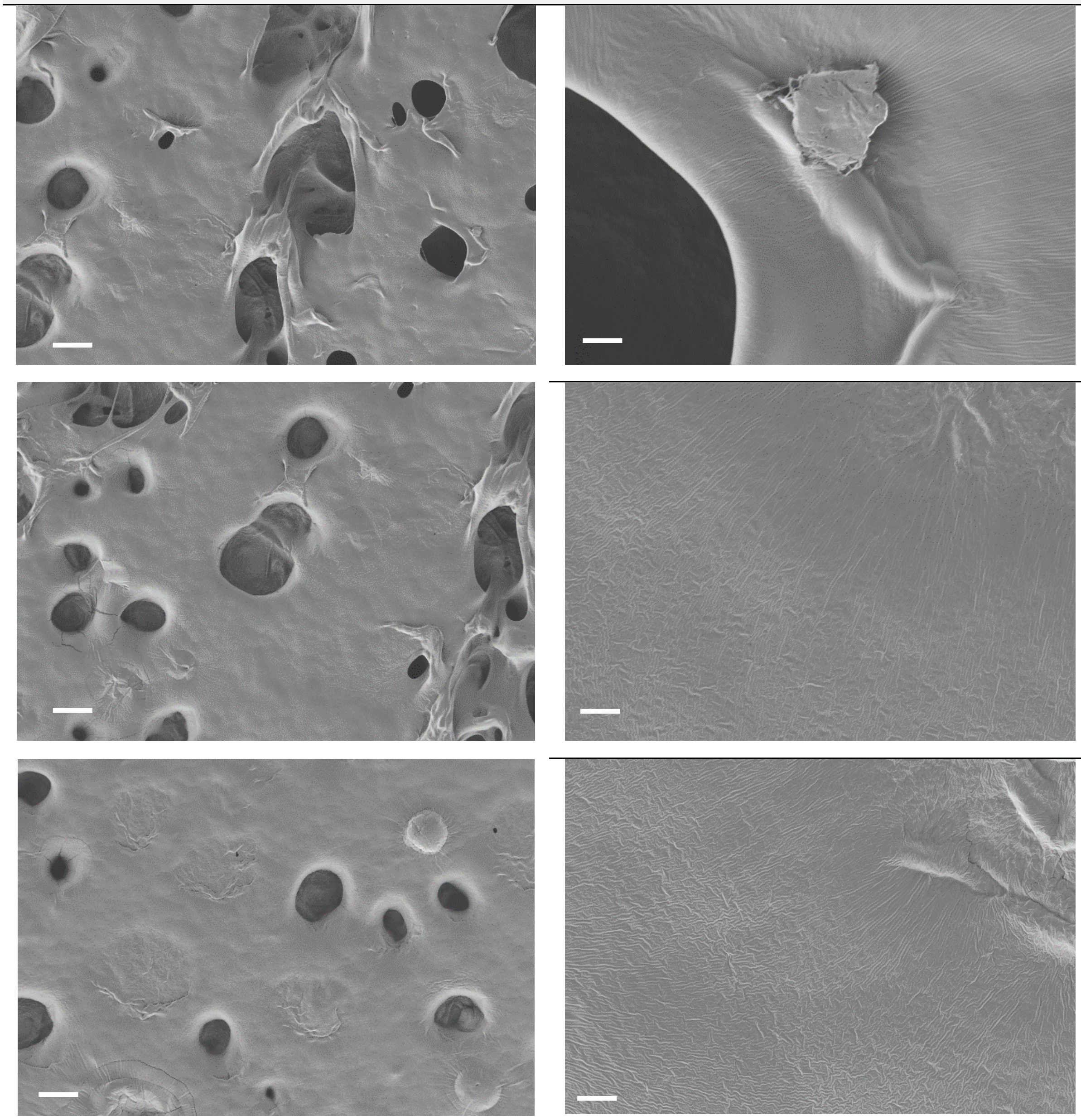

Figure S3. HR-SEM images of the retentate and filtrate of control experiment with 0.45 µm nylon filter paper and only DI (i.e., without raw plastic), illustrating minimal contamination from the filter paper.

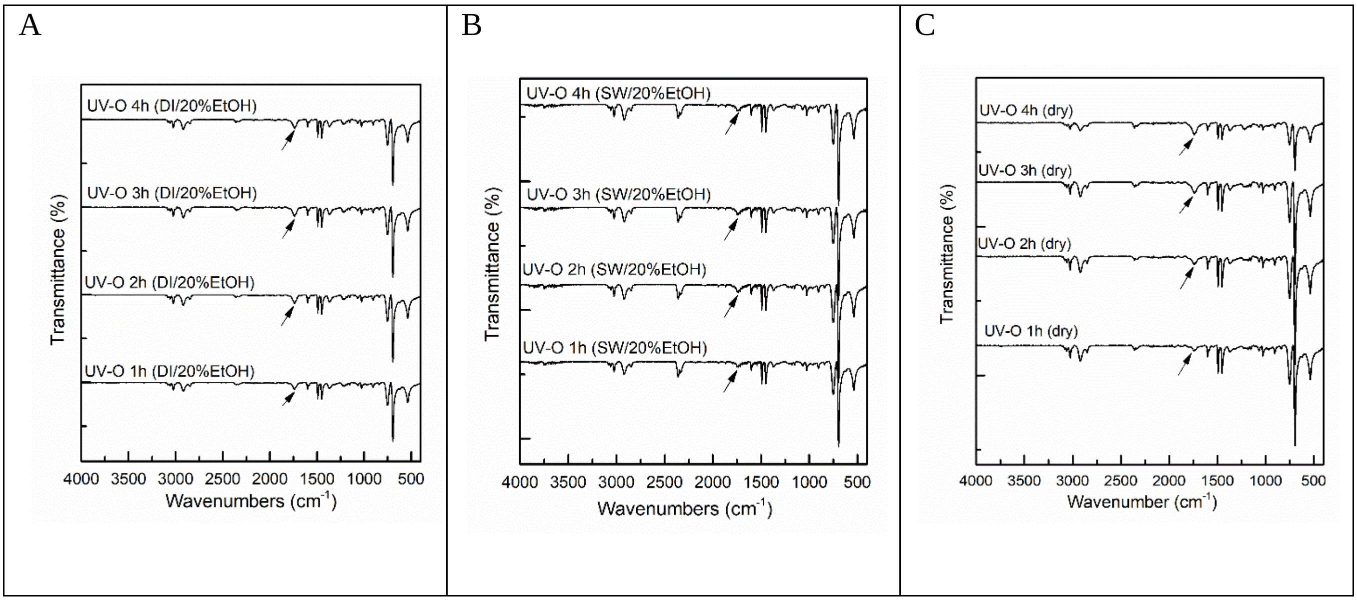

Figure S4. FT-IR of raw plastic treated in a UV-O chamber for one, two, three, and four hours in (A) DI, (B) DI and presence salt to mimic seawater (SW) conditions, and (C) dry conditions; arrows show the generation of carbonyl bond peak at ~1700 cm^{-1}.

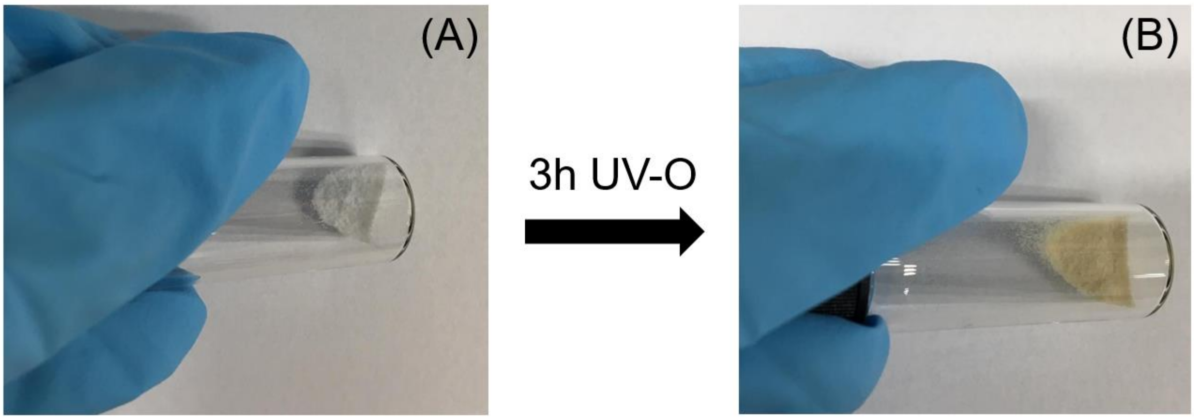

Figure S5. Digital images of UV-O treated raw plastic, confirming the change in color from white (raw plastic) to yellow (oxidized plastic).

Sample's Image	EDX

10 min

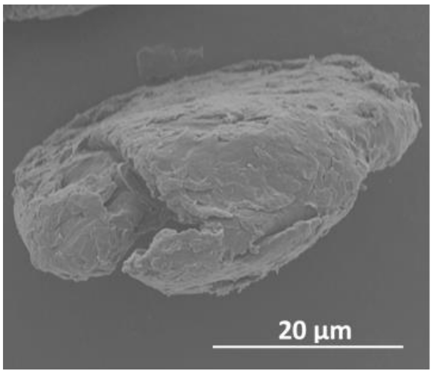

Elements	Weight %
C	93.22
O	6.8
Ti	-

20 min

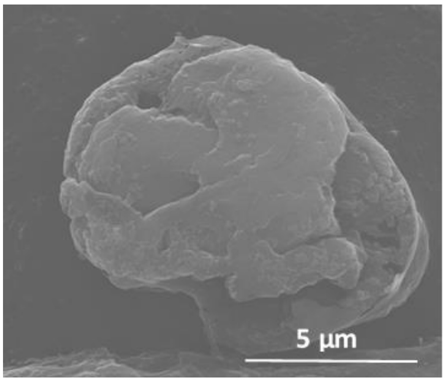

Elements	Weight %
C	92.23
O	7.26
Si	0.31
Ca	0.17
Ti	0.03

30 min

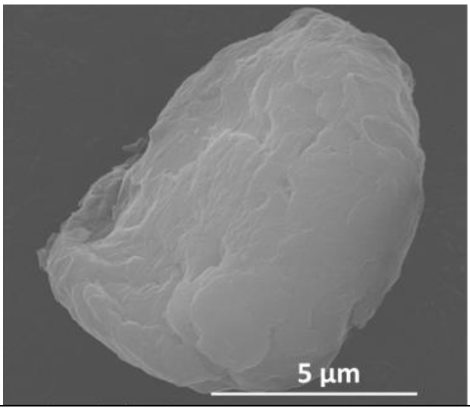

Elements	Weight %
C	99.69
O	0.25
Ti	0.05

Figure S6. EDX for the sonication experiment conducted with the raw plastic under DI/20% EtOH in 10, 20, and 30 minutes.

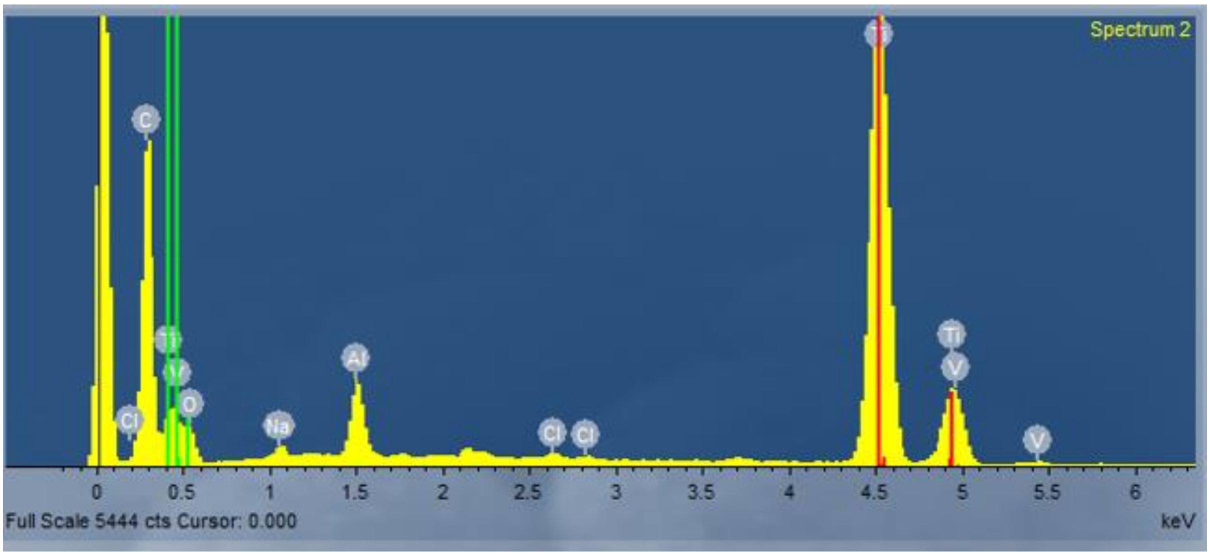

Figure S7. The presence of vanadium, titanium, iron, and aluminium in particles generated during probe sonication as confirmed by EDX in a control experiment (withour using any raw plastic).

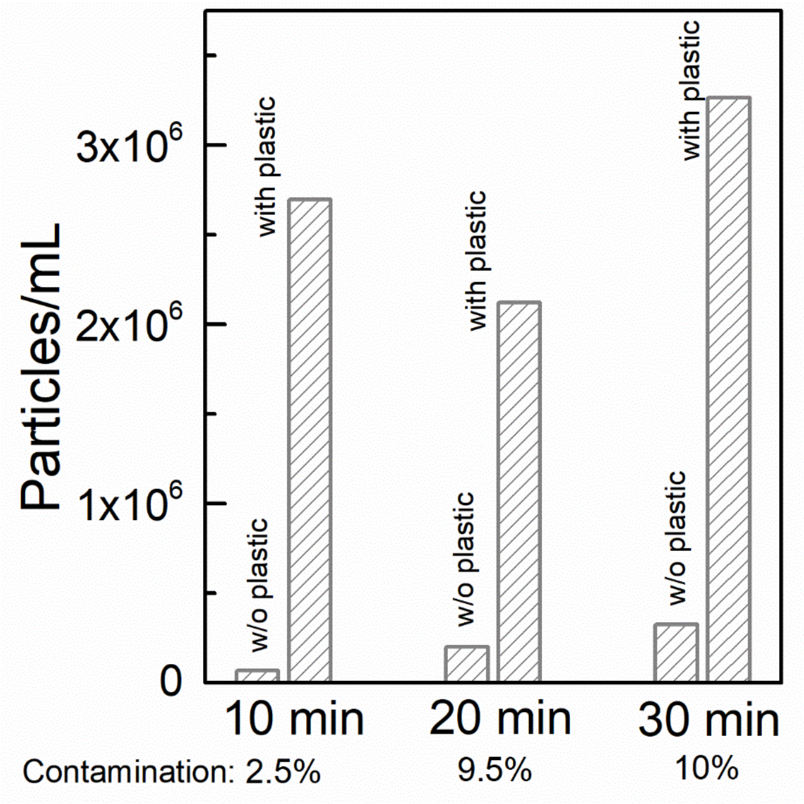

Figure S8. Particle concentration following probe sonication for 10, 20, and 30 minutes with and without raw plastic particles. Results show approximately 10% contamination for sonication times higher than 10 minutes.

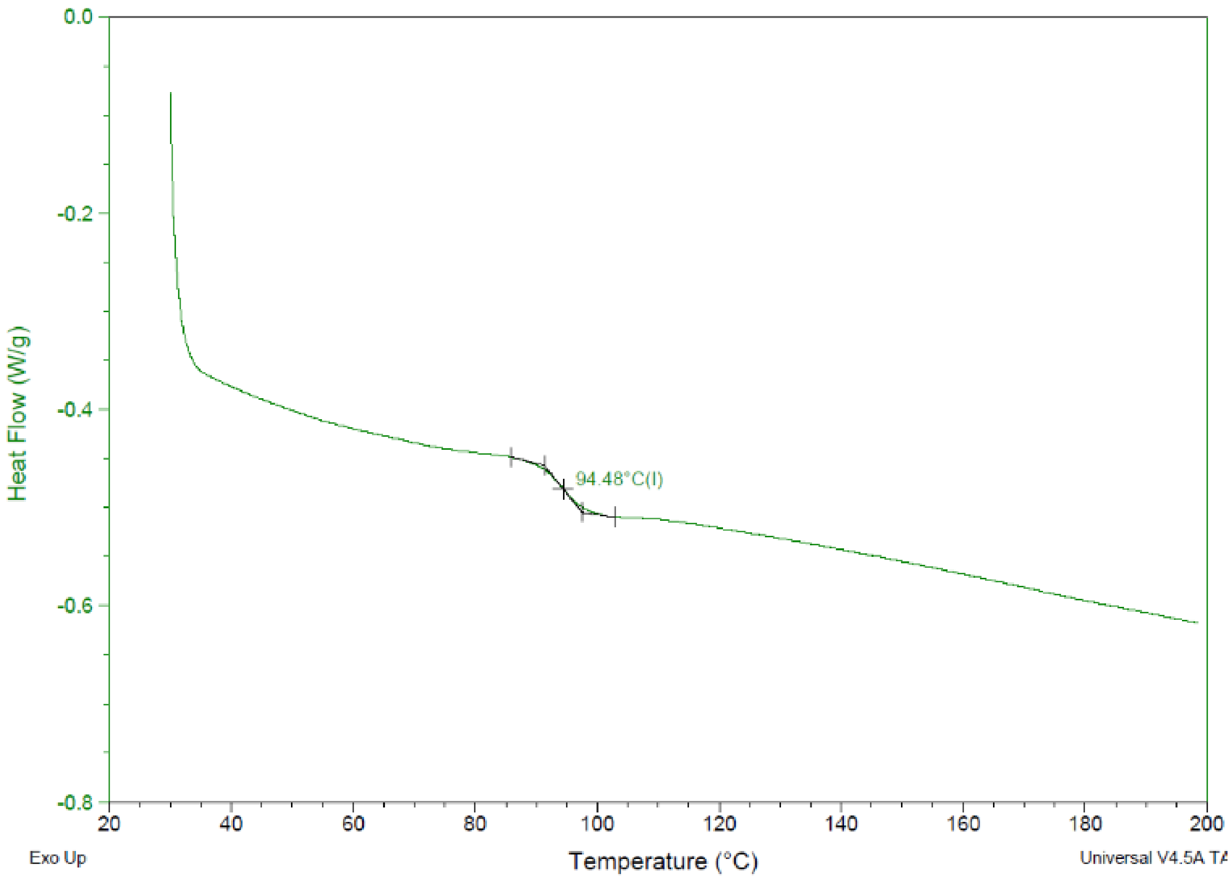

Figure S9. The DSC spectrum shows a glass transition temperature (Tg) of 94.48 °C for raw plastic.

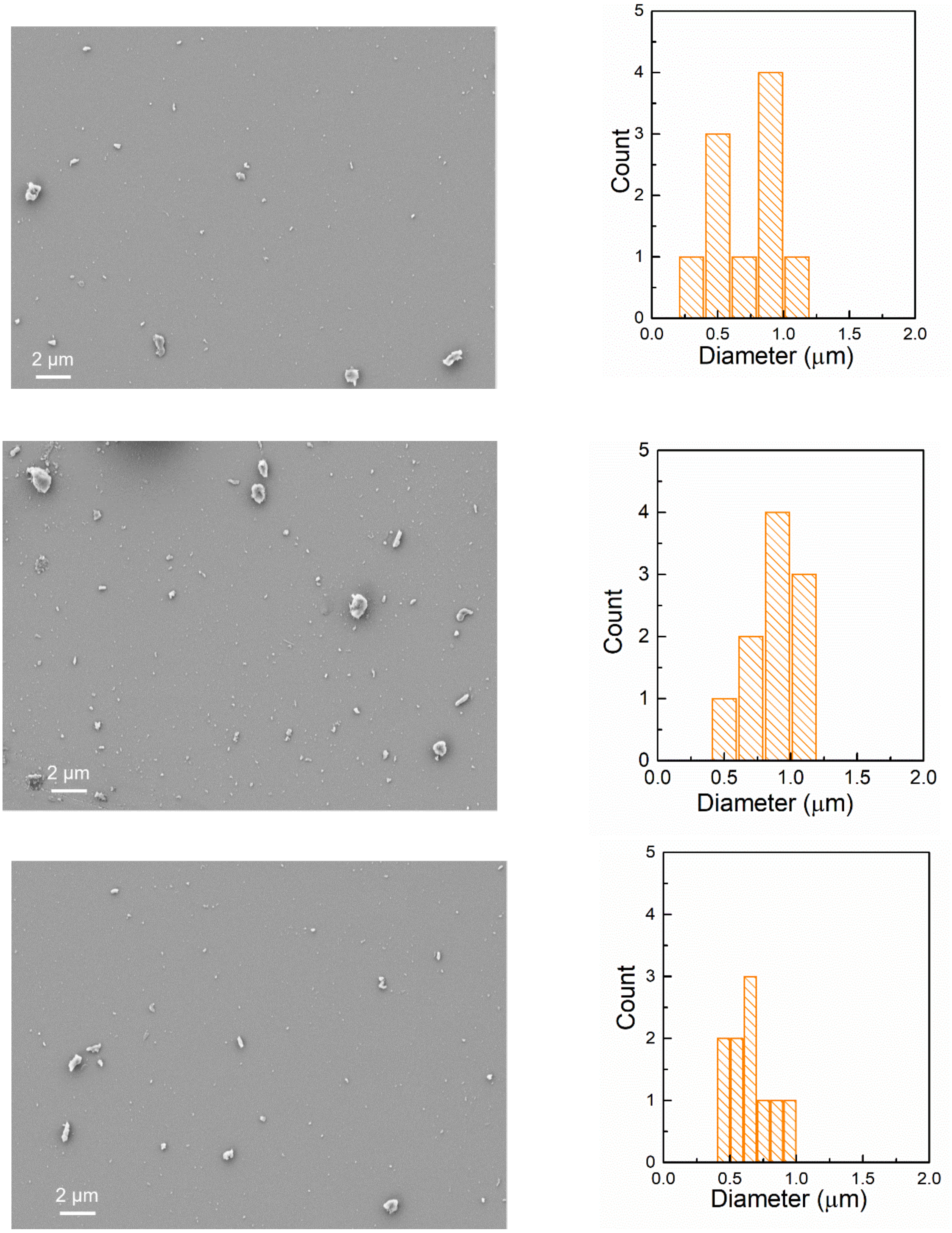

Figure S10. HR-SEM images of engineered MPs and their corresponding particle size distributions calculated using the ImageJ postprocessing software.

Table S1. Characteristics of extracted polystyrene (PS) based environmental microplastics (MPs) from aquatic environments.

Real-environmental PS				
**MP source**	**Size and morphology**	**Main functional groups**	**Other characteristics**	**References**
PS MPs collected at the small islands of Bintan water, Indonesia	1 mm–5 mm	Indications for oxidation through FT-IR analysis (peak appears at ~1650 cm^{-1}); Impurities were detected on MP surface	None	Syakti et al. Mar. Pollut. Bull. 2018[1]
Sediment PS MPs extracted from Santa Barbara Basin bathymetry, USA	MPs were found in the form of fibers, fragments, film, and spherical particles	Indications for oxidation through FT-IR analysis (peak appears at ~1700 cm^{-1}); Contamination was detected on sediment plastics	None	Brandan et al. Sci. Adv. 2019[2]
PS MPs collected from the Geoje island and Nakdong river, South Korea	50 µm–500 µm; <1 mm in beach sand	Indications for oxidation through FT-IR analysis (peak appears at ~1720 cm^{-1})	None	Song et al. Environ. Sci. Technol. 2014 [3]
PS MPs extracted from beach sediments around the Ras Rakan Island of Qatar	Yellowish PS MPs	None	None	Veerasingam et al. Environ Sci Pollut Res. 2021[4]
PS MPs collected from the island beaches in the Pearl River Estuary, China	Rough, irregular, and cracked surface morphologies	None	None	Xie et al. Ecotoxicology 2021[5]
PS plastic debris (foam) collected from the coastal	0.45–1.00 mm Rough and uneven surface morphologies	Indications for oxidation through FT-IR analysis (peak appears at ~3400 cm^{-1})	Specific surface area of ~7.91 m^2/g	Zhang et al. 2018 [6]

beaches of North China				
MPs collected from the shores of the Tuul River, Mongolia	<5 mm; Brownish MPs; Fragmented and rough surface morphologies	Indications for oxidation through FT-IR analysis (peak appears at ~1732 cm^{-1})	Carbonyl index was ranging between 0.00 and 1.09.	Battulga et al. Environ. Pollut. 2020 [7]
PS MPs collected from the Bohai Sea, China	Colored PS-MPs; Rough and fragmented surface morphologies	Indications for oxidation through FT-IR analysis (peak appears at ~1700 cm^{-1})	None	Yu et al. Environ. Pollut. 2016 [8]

(I) (II)

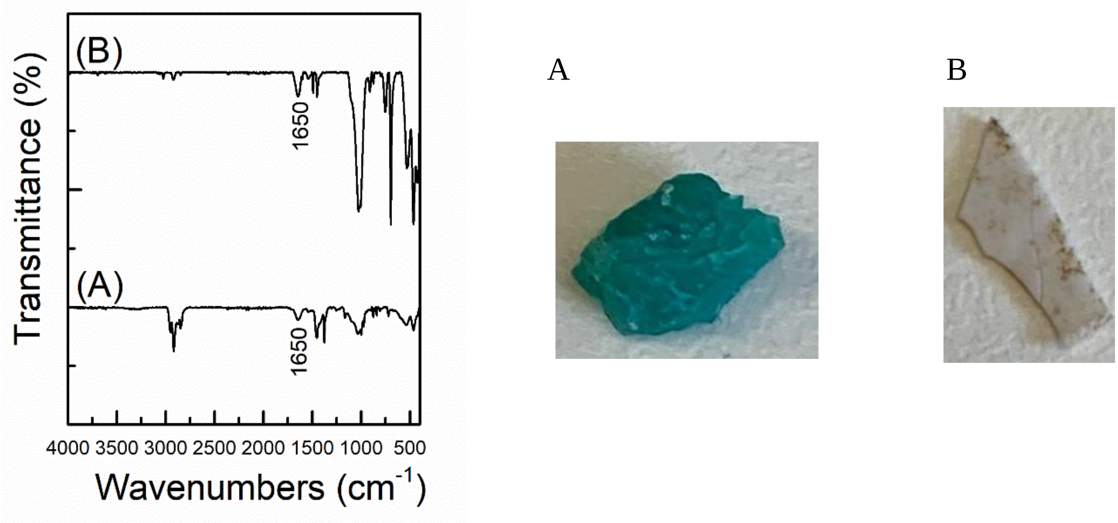

Figure S11. (I) FT-IR spectra and (II) their corresponding digital images of real PS-based environmental plastic collected from Israeli coast of the Mediterranean Sea (Tel Aviv, Israel).

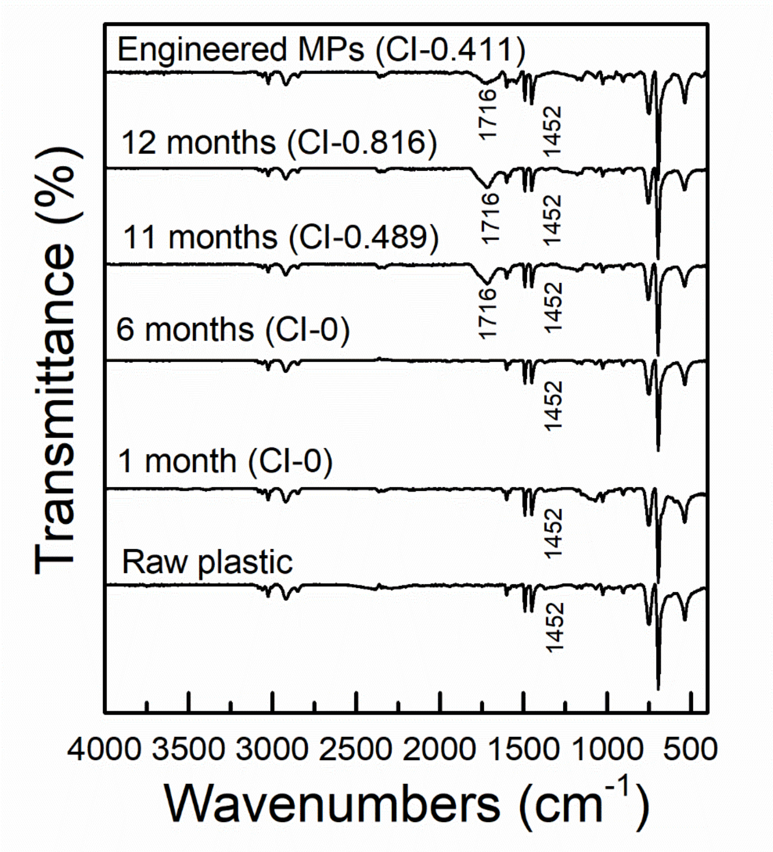

Figure S12. FT-IR spectra of raw plastic, engineered MPs, and semi-environmental plastic at different time (months) for the calculation of carbonyl index (CI).

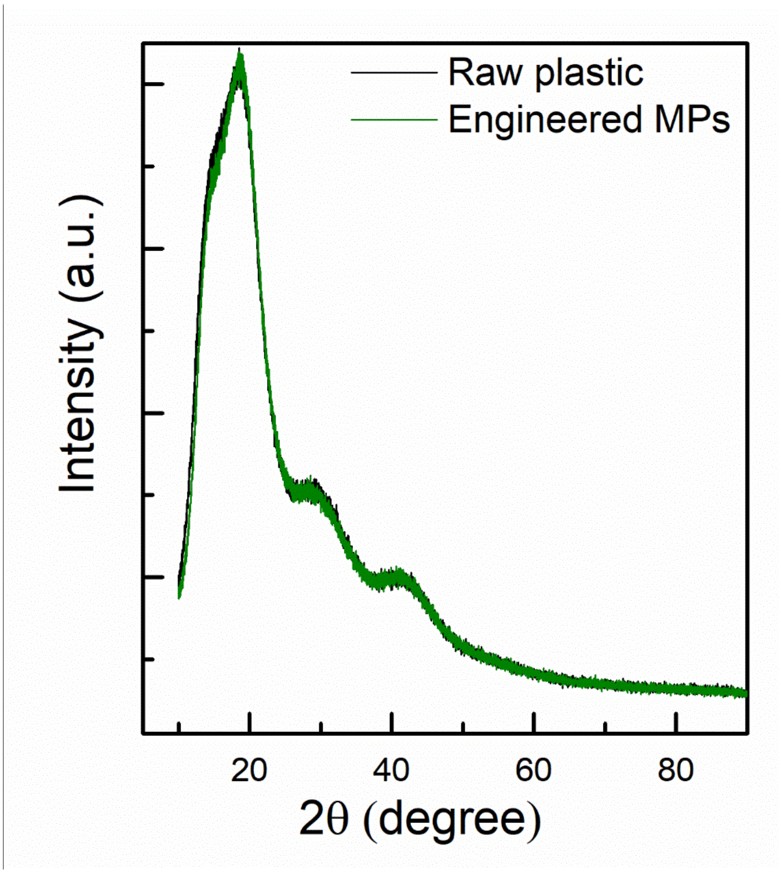

Figure S13. XRD spectra of raw plastic and engineered MPs.

Calculation of energy requirements of single degradation procedures and combined Protocol 3:

Probe sonication technique was performed in order to imitate mechanical degradation. Energy consumption by probe sonication (Ep) can be estimated using **equation S1:**

$$Ep(\frac{J}{L}) = \frac{Wp \times A\% \times t}{V} \quad \text{(S1)}$$

where *Wp* is the sonicator wattage (125 W), *A%* is the amplitude percentage being used in the accelerated procedure (70%), *t* is the sonication time in seconds (600 sec), and *V* is the volume of the solution in liters (0.05L). The calculated energy consumption was divided by the concentration of 1-µm particles yielded during sonication (i.e., particle concentration following subtraction of background particles in raw plastic and particle contamination from probe erosion), to report on energy required to create a single 1-µm particle. The energy consumption by probe sonication at the applied conditions was found to be 1.05×10^6 J/L and energy required to create a single 1-µm particle was 1.66 J/L per particle.

Similarly, we evaluated the energy consumption from heat treatment using **equation S2:**

$$Qp\left(\frac{J}{L}\right) = \frac{m \times c \times \Delta T}{V} \quad \text{(S2)}$$

where *m* is the heated plastic mass in kg (10^{-4} kg), *c* is the specific heat capacity in j/kg C° (1200 j/kg C° for polystyrene),[9] *ΔT* is the temperature differences between oven temperature and room temperature in C° (45°C), and *V* is the volume of 100 mg of the particles in liters (ρPS= 1.04-1.08 gr/cm^2 and equal to 0.0001 L)[10]. Finally, the calculated energy needed to create a single 1-µm particle was 540 J/L per particle, much higher than that of probe sonication. The reason for this large difference between energy consumption for heat treatment and probe sonication is the fact that heat resulted in negligible particle formation compared to probe sonication.

UV-O treatment was used to imitate photo-chemical degradation. The evaluated energy consumption to irradiated area covered with layer of PS particles (Euv) under the suggested setup was calculate in joule (J) per particle (1-µm size) in litter (L) using **equation S3 and S4:**

$$W = \frac{J \times t}{A} \quad \text{(S3)}$$

$$E_{uv}(\frac{J}{L}) = \frac{J}{V} \quad \text{(S4)}$$

where *W* is the instrument power in milliwatts per cm^2 (4.6 mW/cm^2), *A* is the irradiated area in cm^2 (21 cm^2), *t* is the oxidation time in seconds (10800 sec), and *V* is the volume of 100 mg particles in liters (0.0001L). The calculated energy needed to create a single 1-µm particle is 76.05 J/L per particle.

In order to evaluate potential synergistic effect of the combined accelerated oxidation (3h of dry UV-ozone treatment), thermal (12h at 70 °C), and mechanical (10 min of 7 sec on/3 sec off cycles of

probe sonication) procedures, the energetic efficiency of the overall protocol was compared to that of single approaches. The energetic efficiency of protocol 3 was calculated using **equation S5:**

$$En = \frac{\sum E}{Pn} \qquad \text{(S5)}$$

where *n* is the protocol number, *ΣE* is the energy summary of accelerated mechanical, thermal, and photo-oxidation procedures, and *P(n)* is the 1-µm particle count following the degradation protocol. Interestingly, the energy consumption calculated for Protocol 3 was 0.37 J/L per particle, lower than each individual degradation procedures.

References:

(1) Syakti, A. D.; Hidayati, N. V.; Jaya, Y. V.; Siregar, S. H.; Yude, R.; Suhendy; Asia, L.; Wong-Wah-Chung, P.; Doumenq, P. Simultaneous Grading of Microplastic Size Sampling in the Small Islands of Bintan Water, Indonesia. *Mar. Pollut. Bull.* **2018**, *137*, 593–600. https://doi.org/10.1016/j.marpolbul.2018.11.005.

(2) Brandon, J. A.; Jones, W.; Ohman, M. D. Multidecadal Increase in Plastic Particles in Coastal Ocean Sediments. *Sci. Adv.* **2019**, *5* (9), eaax0587. https://doi.org/10.1126/sciadv.aax0587.

(3) Song, Y. K.; Hong, S. H.; Jang, M.; Kang, J.-H.; Kwon, O. Y.; Han, G. M.; Shim, W. J. Large Accumulation of Micro-Sized Synthetic Polymer Particles in the Sea Surface Microlayer. *Environ. Sci. Technol.* **2014**, *48* (16), 9014–9021. https://doi.org/10.1021/es501757s.

(4) Veerasingam, S.; Vethamony, P.; Aboobacker, V. M.; Giraldes, A. E.; Dib, S.; Al-Khayat, J. A. Factors Influencing the Vertical Distribution of Microplastics in the Beach Sediments around the Ras Rakan Island, Qatar. *Environ. Sci. Pollut. Res.* **2021**. https://doi.org/10.1007/s11356-020-12100-4.

(5) Xie, Q.; Li, H.-X.; Lin, L.; Li, Z.-L.; Huang, J.; Xu, X.-R. Characteristics of Expanded Polystyrene Microplastics on Island Beaches in the Pearl River Estuary: Abundance, Size, Surface Texture and Their Metals-Carrying Capacity. *Ecotoxicology* **2021**. https://doi.org/10.1007/s10646-020-02329-7.

(6) Zhang, H.; Wang, J.; Zhou, B.; Zhou, Y.; Dai, Z.; Zhou, Q.; Chriestie, P.; Luo, Y. Enhanced Adsorption of Oxytetracycline to Weathered Microplastic Polystyrene: Kinetics, Isotherms and Influencing Factors. *Environ. Pollut.* **2018**, *243*, 1550–1557. https://doi.org/https://doi.org/10.1016/j.envpol.2018.09.122.

(7) Battulga, B.; Kawahigashi, M.; Oyuntsetseg, B. Behavior and Distribution of Polystyrene Foams on the Shore of Tuul River in Mongolia. *Environ. Pollut.* **2020**, *260*, 113979. https://doi.org/10.1016/j.envpol.2020.113979.

(8) Yu, X.; Peng, J.; Wang, J.; Wang, K.; Bao, S. Occurrence of Microplastics in the Beach Sand of the Chinese Inner Sea: The Bohai Sea. *Environ. Pollut.* **2016**, *214*, 722–730. https://doi.org/10.1016/j.envpol.2016.04.080.

(9) Lakatos, Á.; Kalmár, F. Investigation of Thickness and Density Dependence of Thermal Conductivity of Expanded Polystyrene Insulation Materials. *Mater. Struct. Constr.* **2013**, *46* (7), 1101–1105. https://doi.org/10.1617/s11527-012-9956-5.

(10) Han, X.; Lu, X.; Vogt, R. D. An Optimized Density-Based Approach for Extracting Microplastics from Soil and Sediment Samples. *Environ. Pollut.* **2019**, *254*, 113009. https://doi.org/10.1016/j.envpol.2019.113009.
